# Minor Protease Inhibitor Mutations at Baseline Do Not Increase the Risk for a Virological Failure in HIV-1 Subtype B Infected Patients

**DOI:** 10.1371/journal.pone.0037983

**Published:** 2012-06-18

**Authors:** Alexandra U. Scherrer, Bruno Ledergerber, Viktor von Wyl, Jürg Böni, Sabine Yerly, Thomas Klimkait, Cristina Cellerai, Hansjakob Furrer, Alexandra Calmy, Matthias Cavassini, Luigia Elzi, Pietro L. Vernazza, Enos Bernasconi, Huldrych F. Günthard, and the Swiss HIV Cohort Study (SHCS)

**Affiliations:** 1 Division of Infectious Diseases and Hospital Epidemiology, University Hospital Zürich, University of Zürich, Zürich, Switzerland; 2 Institute of Medical Virology, Swiss National Center for Retroviruses, University of Zürich, Zürich, Switzerland; 3 Laboratory of Virology, Geneva University Hospitals, Geneva, Switzerland; 4 Institute for Medical Microbiology, University of Basel, Basel, Switzerland; 5 Division of Immunology and Allergy, University Hospital Lausanne, Lausanne, Switzerland; 6 Division of Infectious Diseases, University Hospital Berne, University of Berne, Berne, Switzerland; 7 Division of Infectious Diseases, University Hospitals Geneva, Geneva, Switzerland; 8 Infectious Diseases Service, University Hospital Lausanne, Lausanne, Switzerland; 9 Division of Infectious Diseases and Hospital Epidemiology, University Hospital Basel, Basel, Switzerland; 10 Division of Infectious Diseases, Cantonal Hospital St. Gallen, St. Gallen, Switzerland; 11 Division of Infectious Diseases, Regional Hospital Lugano, Lugano, Switzerland; Centro de Biología Molecular Severo Ochoa (CSIC-UAM), Spain

## Abstract

**Background:**

Minor protease inhibitor (PI) mutations often exist as polymorphisms in HIV-1 sequences from treatment-naïve patients. Previous studies showed that their presence impairs the antiretroviral treatment (ART) response. Evaluating these findings in a larger cohort is essential.

**Methods:**

To study the impact of minor PI mutations on time to viral suppression and time to virological failure, we included patients from the Swiss HIV Cohort Study infected with HIV-1 subtype B who started first-line ART with a PI and two nucleoside reverse transcriptase inhibitors. Cox regression models were performed to compare the outcomes among patients with 0 and ≥1 minor PI mutation. Models were adjusted for baseline HIV-1 RNA, CD4 cell count, sex, transmission category, age, ethnicity, year of ART start, the presence of nucleoside reverse transcriptase inhibitor mutations, and stratified for the administered PIs.

**Results:**

We included 1199 patients of whom 944 (78.7%) received a boosted PI. Minor PI mutations associated with the administered PI were common: 41.7%, 16.1%, 4.7% and 1.9% had 1, 2, 3 or ≥4 mutations, respectively. The time to viral suppression was similar between patients with 0 (reference) and ≥1 minor PI mutation (multivariable hazard ratio (HR): 1.1 [95% confidence interval (CI): 1.0–1.3], *P* = .196). The time to virological failure was also similar (multivariable HR:.9 [95% CI:.5–1.6], *P* = .765). In addition, the impact of each single minor PI mutation was analyzed separately: none was significantly associated with the treatment outcome.

**Conclusions:**

The presence of minor PI mutations at baseline has no effect on the therapy outcome in HIV infected individuals.

## Introduction

Minor protease inhibitor (PI) mutations are very common among treatment-naïve patients infected with HIV-1 but their impact on treatment outcome is poorly understood [1], [2], [3], [4], [5], [6]. The prevalence of different minor PI mutations among treatment-naïve patients varies largely and is highly dependent on the HIV-1 subtype [7], [8], [9]. Some minor PI mutations occur as natural polymorphisms whereas others do not occur in the absence of PI therapy [10]. Minor PI mutations do not lead to high level resistance when occurring alone but they either improve the viral fitness or increase the drug resistance level in the presence of major PI mutations [11], [12]. Minor PI mutations are therefore also called secondary or accessory mutations [13].

It was assumed that minor PI mutations among treatment-naïve patients might facilitate the emergence of major PI mutations and therefore lead to a worse therapeutic response to PIs. Other studies analyzing this issue were quite controversial. Perno et al. found evidence that the presence of minor PI mutations, particularly at position 10 and 36, lead to early treatment failure and to a higher number of acquired major PI mutations at the time of treatment failure [14], [15]. Other studies found no evidence for an impaired treatment outcome [16], [17], [18], [19]. All these studies are limited by a rather small sample size and mainly focus on response to unboosted PI therapy which is no longer recommended [20].

Therefore, we aimed studying the impact of minor PI mutations on virological outcome in first-line antiretroviral therapy (ART) using the dataset of the Swiss HIV Cohort Study (SHCS) [21].

## Methods

### Ethics Statement

The SHCS has been approved by the following ethical committees of all participating institutions: Kantonale Ethikkommission Bern; Ethikkommission beider Basel; comité d’éthique du département de médicine de Hôpitaux Universitaires de Genève; commission d’éthique de la recherche clinique, Lausanne; comitato etico cantonale, Bellinzona; Ethikkommission des Kanton St.Gallens; and Ethik-Kommission Zürich, all Switzerland. Written informed consent has been obtained from all participants.

### Study Population

We used data from the SHCS, a nationwide, multicenter, clinic-based cohort with continuous enrolment and semi-annual study visits. Data up to 13 September 2011 were considered. The SHCS is very representative and includes about 66% of patients living with AIDS in Switzerland and 75% of all patients receiving antiretroviral therapy [21]. In addition, we used data from the SHCS drug resistance database that includes sequences from all authorized laboratories in Switzerland. Sequences are stored in SmartGene’s (Zug, Switzerland) Integrated Database Network System (IDNS version 3.6.5) [22].

**Table 1:** Baseline characteristics.

Characteristics	≥1 minor PI mutation	No minor PI mutation	*P* *
Sex			.227
Male	640 (82.9%)	342 (80.1%)	
Female	132 (17.1%)	85 (19.9%)	
Ethnicity			.149
White	701 (90.8%)	398 (93.2%)	
Other	71 (9.2%)	29 (6.8%)	
Transmission category			.020
Men who have sex with men	426 (55.2%)	213 (49.9%)	
Heterosexual	187 (24.2%)	111 (26.0%)	
Intravenous drug use	129 (16.7%)	95 (22.3%)	
Other	30 (3.9%)	8 (1.9%)	
Median [IQR] age	45 [39–51]	45 [39–51]	.984
HIV-1 RNA			.263
<10,000 copies/mL	133 (17.2%)	70 (16.4%)	
10,000–99,999 copies/mL	278 (36.0%)	174 (40.8%)	
>100000 copies/mL	361 (46.8%)	183 (42.9%)	
Median [IQR] log_10_ HIV-1 RNA	4.9 [4.4–5.5]	4.9 [4.3–5.4]	.315
CD4 cell count			.024
<200 cells/µL	334 (43.3%)	156 (36.5%)	
200–300 cells/µL	253 (32.8%)	141 (33.0%)	
>350 cells/µL	185 (24.0%)	130 (30.4%)	
Median (IQR) CD4 cells/µL	223 [125.5–339.5]	255 [141–379]	.010
CDC stage C	137 (17.8%)	65 (15.2%)	.264
NRTI mutation	33 (4.3%)	12 (2.8%)	.201
Administered PIs			<.001
unboosted PIs	136 (17.6%)	119 (27.9%)	
boosted PIs	636 (82.4%)	308 (72.1%)	
Specific PI			<.001
Nelfinavir	115 (14.9%)	105 (24.6%)	
Other unboosted PIs	21 (2.7%)	14 (3.3%)	
Lopinavir	415 (53.8%)	175 (41.0%)	
Atazanavir/r	193 (25.0%)	53 (12.4%)	
Indinavir/r	23 (3.0%)	20 (4.7%)	
Other boosted PIs	5 (0.7%)	60 (14.1%)	
Median [IQR] year of ART start	2006 [2003–2008]	2005 [2001–2008]	.005

P* Fishers exact p value for categorical variable and Wilcoxon rank sum for continuous variables.

Abbreviations: ART, antiretroviral therapy; CDC, Centers for Disease Control and Prevention; IQR, interquartile range; NRTI nucleoside reverse transcriptase inhibitor; PI, protease inhibitor.

### Patient Selection and Study Design

We included HIV-1 subtype B infected individuals who started first-line ART between 1 January 1999 and 1 July 2010 with an unboosted PI or a boosted PI and two nucleoside reverse transcriptase inhibitors (NRTIs) and who had CD4 cell counts and HIV-1 plasma RNA levels measured before start of ART. A genotypic resistance test performed while ART-naïve was an additional inclusion criterion. Patients were excluded if they had viruses with ≥1 transmitted major PI mutation or if they had no HIV-1 RNA measured during first-line ART [23].

We studied the following endpoints: a) time to viral suppression, b) time to virological failure, and c) accumulation of major mutations at the time of virological failure. Time to viral suppression was defined as the time to the first viral load <50 copies/mL. Virological failure was defined as 2 consecutive values >500 copies/mL after at least 180 days of continuous treatment, 1 value >500 after 180 days followed by a treatment change or no viral suppression for more than 180 days. To fulfill the criteria of a virological failure, patients needed a minimum time of follow-up, therefore the analysis of time to virological failure was restricted to patients with ≥1 HIV-1 RNA measurement after 180 days of continuous treatment or to patients with ≥1 HIV-1 RNA measurement after previous viral suppression. The accumulation of major mutations at virological failure was studied in patients who experienced a virological failure on first-line ART and who had a genotypic resistance test performed between the virological failure and treatment change.

Minor PI mutations were defined based on the IAS-USA recommendations [23]. In the following we term mutations as related to a specific drug if they are listed as minor PI mutations on the IAS-USA drug resistance mutation list [23]. Minor PI mutations related to the following PIs were analyzed: atazanavir (L10I/F/V/C, G16E, K20R/M/I/T/V, L24I, V32I, L33I/F/V, E34Q, M36I/L/V, M46I/L,G48V, F53L/Y, I54L/V/M/T/A, D60E, I62V, I64L/M/V, A71V/I/T/L,G73C/S/T/A, V82A/T/F/I, I85V, L90M, I93L/M), darunavir (V11I, V32I, L33F, T74P, L89V), fosamprenavir (L10F/I/R/V, V32I, M46I/L, I47V, I54L/V/M, G73S, L76V, V82A/F/S/T, L90M), indinavir (L10I/R/V, K20M/R, L24I, V32I, M36I, I54V, A71V/T, G73S/A, L76V, V77I, L90M), lopinavir (L10F/I/R/V, K20M/R, L24I, L33F, M46I/L, I50V, F53L, I54V/L/A/M/T/S, L63P, A71V/T, G73S, I84V, L90M), nelfinavir (L10F/I, M36I, M46I/L, A71V/T, V77I, V82A/F/T/S, I84V, N88D/S) and saquinavir (L10I/R/V, L24I, I54V/L, I62V, A71V/T, G73S, V77I, V82A/F/T/S, I84V). No patient was treated with tipranavir.

**Figure 1:**
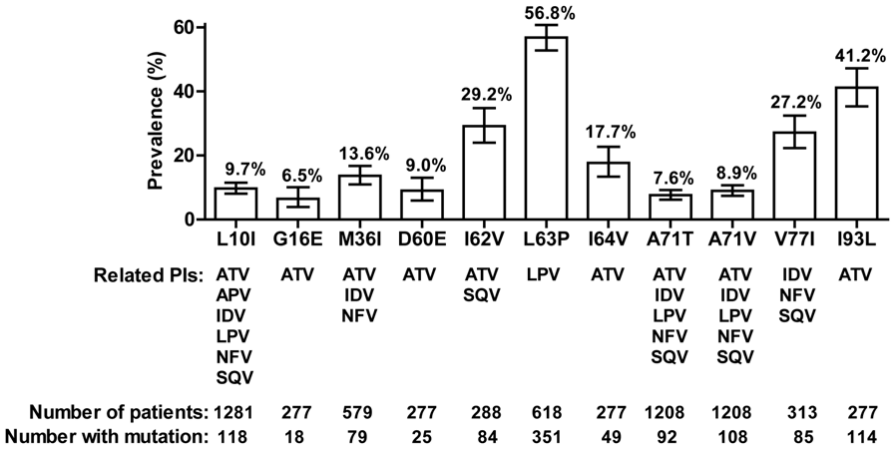
Prevalence of minor protease inhibitor (PI) mutations. Minor PI mutations with a prevalence ≥5% and PI treatments potentially related to these mutations [22]. Error bars represent 95% confidence interval.

### Statistical Analysis

We performed Fisher’s exact tests and Wilcoxon rank sum tests to compare categorical and continuous baseline characteristics, respectively. We plotted Kaplan-Meier curves and used log-rank tests to compare the virological outcome between patients with and without minor PI mutations. In addition, we performed univariable and multivariable Cox regression to analyze the time to viral suppression and the time to virological failure. Multivariable models were adjusted for the following potential confounders: sex, ethnicity, age, transmission category, baseline CD4 cell count, baseline HIV-1 RNA level, calendar year of ART start and the presence of NRTI mutations [23] and stratified for the PI used. Continuous variables were categorized if likelihood ratio tests showed significant departure from linearity. Follow-up was censored when first-line ART was changed or stopped. We checked the proportional hazard assumption with Schoenfeld residuals and by using graphical methods. No violation was found.

We also studied the impact of specific minor PI mutations on virological outcome. Here, only mutations with a prevalence ≥5% were considered. Despite this restriction, the number of events for some mutations was quite small, particularly the number of virological failures. Therefore, we used other methods that can deal better with rare events. It was shown that propensity scores are a good alternative to control for imbalances between groups when there are only small numbers of events per confounder [24]. In a 2-step procedure, we first calculated for each patient the propensity of being in the group with or without minor PI mutation. This was done by calculating propensity scores with multivariable logistic regression models adjusted for baseline HIV-1 RNA level, baseline CD4 cell count, ethnicity, sex, transmission category, calendar year of ART start, presence of NRTI mutations and the PI used. We validated if the propensity scores balanced the differences between groups adequately. Therefore, we performed logistic regression models adjusted for the propensity score to test if there were still imbalanced co-variables that were significantly associated with a group after adjustment. No poorly balanced co-variables were found. We did not use c statistics for model building of propensity score methods because it might be inadequate [25], [26]. In a second step, we used the propensity scores for regression adjustment. The virological outcomes were analyzed with a Cox regression models adjusted for the log-transformed propensity score as the single co-variable. The log transformation is necessary for the adjustment because the variance of propensity scores needs to be similar between patients with and without minor PI mutations [27], [28].

The accumulation of major PI mutations at the time of virological failure was compared with Fisher’s exact test.

Statistical analyses were performed with Stata 11 (StataCorp, College Station, TX).

**Figure 2:**
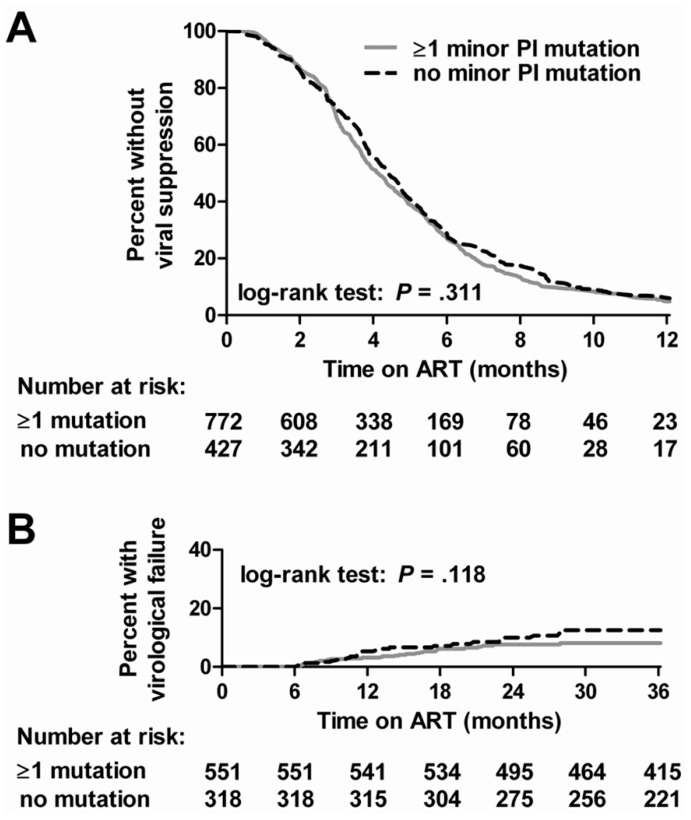
Kaplan-Meier curves. Kaplan-Meier curves comparing (A) time to viral suppression and (B) time to virological failure between patients with ≥1 and without minor protease inhibitor (PI) mutations detected.

**Table 2:** Cox regression models analyzing time to viral suppression and time to virological failure. Models were stratified for the administered PI.

	Viral Suppression					Virological failure			
Characteristics	univariable HR(95% CI)	*P*	multivariable HR (95% CI)	*P*		univariable HR(95% CI)	*P*	multivariable HR(95% CI)	*P*
Minor PI mutation									
No	Ref		Ref			Ref		Ref	
Yes	1.1 (0.9–1.2)	.364	1.1 (1.0–1.3)	.196		1.0 (0.6–1.6)	.935	0.9 (0.5–1.6)	.765
Sex									
Male	Ref		Ref			Ref		Ref	
Female	1.3 (1.1–1.6)	.001	1.3 (1.1–1.5)	.003		0.6 (0.3–1.3)	.188	0.5 (0.2–1.2)	.142
Ethnicity									
White	Ref		Ref			Ref		Ref	
Other	1.3 (1.0–1.6)	.020	1.1 (0.9–1.5)	.250		1.2 (0.5–3.0)	.724	1.2 (0.5–3.3)	.690
Age (per 10 years)	1.0 (0.9–1.0)	.366	1.0 (0.9–1.1)	.670		0.9 (0.7–1.2)	.363	0.9 (0.6–1.2)	.374
Transmission category									
Other	Ref		Ref			Ref		Ref	
IDU	1.0 (0.8–1.2)	.806	0.9 (0.7–1.1)	.188		1.2 (0.7–2.0)	.551	1.5 (0.9–2.7)	.145
CD4 cell count									
<200 cells/µl	Ref		Ref			Ref		Ref	
200–350 cells/µl	1.2 (1.1–1.4)	.009	1.1 (1.0–1.3)	.103		0.7 (0.3–1.2)	.170	0.6 (0.3–1.2)	.125
>350 cells/µl	1.4 (1.2–1.7)	<.001	1.3 (1.1–1.6)	.001		1.2 (0.7–2.3)	.496	1.3 (0.6–2.5)	.500
HIV-1 RNA									
<10,000 copies/mL	Ref		Ref			Ref		Ref	
10,000–99,999 copies/mL	0.7 (0.6–0.8)	<.001	0.7 (0.6–0.9)	.001		1.3 (0.6–3.0)	.513	1.6 (0.7–4.0)	.279
>100,000 copies/mL	0.4 (0.4–0.5)	<.001	0.5 (0.4–0.5)	<.001		1.6 (0.7–3.7)	.259	2.3 (0.9–5.8)	.081
Year of ART start	1.0 (1.0–1.0)	.321	1.0 (1.0–1.0)	.325		0.9 (0.8–1.1)	.536	1.0 (0.8–1.2)	.994
NRTI mutation									
No	Ref		Ref			Ref		Ref	
Yes	1.0 (0.7–1.4)	.828	0.7 (0.5–1.1)	.106		3.7 (1.6–8.5)	.002	6.1 (2.5–15.0)	<.001

Abbreviations: HR, hazard ratio; CI, confidence interval; PI, protease inhibitor; ART, antiretroviral therapy; NRTI, nucleoside reverse transcriptase inhibitor.

## Results

### Study Population and Baseline Characteristics

In the SHCS, 1265 subtype B-infected patients started first-line ART with a PI and 2 NRTIs and had a resistance test performed while ART-naïve. Patients were excluded from analysis if they had major PI mutations detected (n = 1), missing baseline HIV-1 RNA levels or CD4 cell counts (n = 14), or no HIV-1 RNA follow-up before the first ART change (n = 51). Finally, 1199 of 1265 patients (94.8%) were included to study the time to viral suppression. In table 1, we showed the baseline characteristics. Minor PI mutations were highly prevalent and present among 772 (64.4%) patients. Slightly more patients with a minor PI mutation were treated with a boosted PI, 82.4% compared to 72.1% without minor PI mutation (*P*<.001).The median CD4 cell count was higher among patients without minor PI mutation, 255 cells/µL compared to 223 cells/µL (*P* = .010). The most common NRTI combinations were lamivudine/zidovudine (43.3%), emtricitabine/tenofovir (33.2%) and lamivudine/abacavir (9.6%), no differences were observed between patients with and without minor PI mutations. 869 patients (72.5%) had the required minimum follow-up time to study the time to virological failure. Baseline characteristics of excluded patients did not markedly differ except that excluded patients started ART earlier (median: 2005 compared to 2006, *P* = .006).

**Figure 3:**
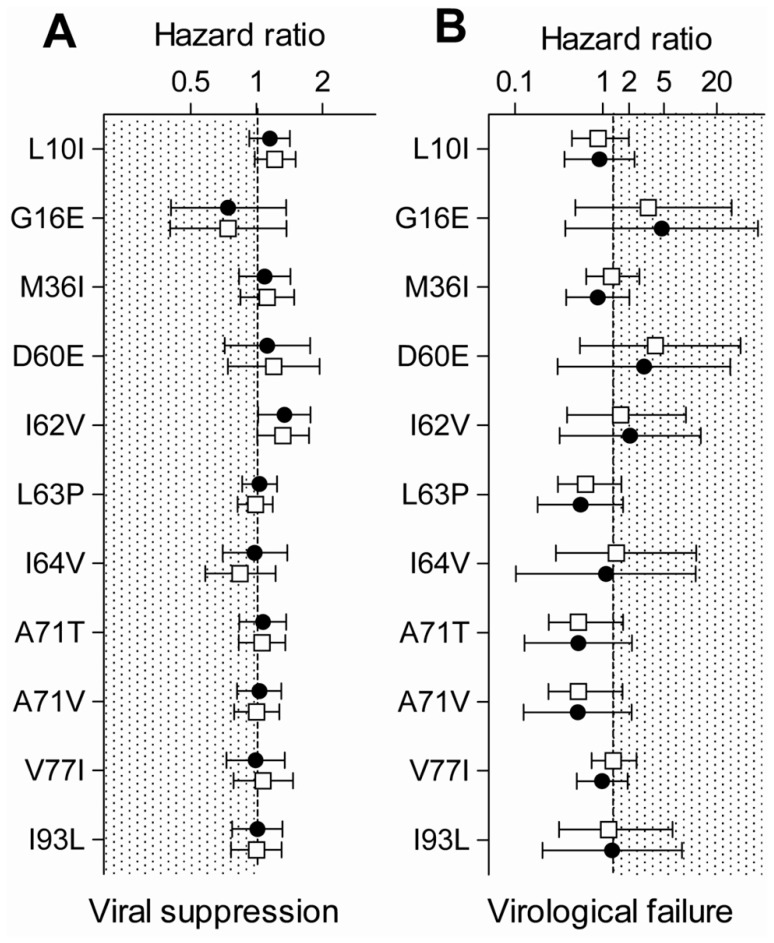
Cox regression models. Univariable (solid circles) and multivariable (open squares) Cox regression models comparing (A) time to viral suppression and (B) time to virological failure. 95% confidence intervals are indicated. The treatment outcome is worse in the presence of minor protease inhibitor mutations if the hazard ratios lie in the shaded area.

### Prevalence of Specific Minor PI Mutations

The prevalence of the most common minor PI mutations related the respective administered PI therapies is shown in Figure 1. L63P was the most common minor PI mutation, it was present among 351 of 618 (56.8%) patients before treatment with lopinavir. Followed by the atazanavir related mutation I93L (41.2%, n = 114/277), the atazanavir/saquinavir related mutation I62V (n = 84/288, 29.2%) and the indinavir/nelfinavir/saquinavir related mutation V77I (n = 85/313, 27.2%). L10I and M36I were found to be associated with a worse treatment in previous studies [14], [15]. In our study, they occurred in 9.7% (n = 118/1218) and 13.6% (n = 79/579) samples, respectively. The following mutations had a prevalence of <5%: L10F (0.2%)/R (0%)/V(1.9%), V11I (0%), K20I (0%)/M (0.4%)/R (2.3%)/T (0%)/V (0%), L24I (0.1%), V32I (0%), L33F (0.4%)/I (1.4%)/V (2.9%), E34Q (0%), E36L (1.1%)/V (0.4%), M46I (0.2%)/L (0%), I47V (0%), G48V (0%), I50V (0%), F53L (0%)/Y (0%), I54A (0%)/L (0%)/M (0%)/S (0%)/T (0%)/V (0.2%), I64M (2.5%)/L (2.5%), A71I (0%)/L (0%), G73A (0%)/C (0%)/S (0%)/T (0%), T74P (0%), V82A (0.2%)/F (0%)/I (1.1%)/S (0%)/T (0.2%), I84V (0%), L90M (1.0%), and I93M (0%). Overall, 41.7%, 16.1%, 4.7% and 1.9% of patients had 1, 2, 3 and ≥4 minor PI mutations related to first-line ART.

### Virological Outcome

The time to viral suppression and the time to virological failure were similar between patients with and without minor PI mutations (Figure 2). Results of log-rank tests suggested no relevant differences. As shown in Table 2, univariable and multivariable hazard ratios (HR) were 1.1 (95% CI:.9–1.2) and 1.1 (95% CI: 1.0–1.3) when comparing the time to viral suppression between patients with and without minor PI mutations. A HR below 1 would indicate a longer time to viral suppression among patients carrying viruses with a minor PI mutation. Also the time to virological failure was not significantly different between patients with and without minor PI mutations, univariable and multivariable HRs were 1.0 (95% CI: 0.6–1.9) and 0.9 (95% CI: 0.5–1.6), respectively. The risk for a virological failure would be increased among patients detected with a minor PI mutation if the HR was above 1.

Additionally, we studied the impact of the number of minor PI mutations on the virological outcome. Compared to patients without minor PI mutations, HRs of the time to viral suppression were 1.1 (95% CI: 0.9–1.2), 1.1 (95% CI: 0.9–1.4), 1.5 (95% CI: 1.1–2.1) and 1.4 (95% CI: 0.9–2.3) for patients with 1, 2, 3, ≥4 minor PI mutations, respectively. For the time to virological failure HRs were 1.1 (95% CI: 0.6–1.8), 0.5 (95% CI: 0.2–1.5), 0.3 (0.1–3.8) and 1.0 (0.1–10.3) for patients with 1, 2, 3, ≥4 minor PI mutations, respectively. Comparing patients with <3 and ≥3 minor PI mutations did not alter conclusions, the HR for the time to viral suppression was 1.2 (95% CI: 0.9–1.4) and for the time to virological failure 0.6 (95% CI: 0.2–2.0).

We additionally studied the effect of specific minor PI mutations on the virological outcome. No specific minor PI mutation was associated with a worse treatment outcome (Figure 3). We studied all minor PI mutations with a prevalence ≥5%. The 95% confidence interval of HRs always included 1, meaning that no significant differences were observed between patients with and without minor PI mutations.

We performed a sensitivity analyses and ran separate models for ART with unboosted PI and boosted PIs. For the time to viral suppression, multivariable HRs were 1.0 (95% CI: 0.6–1.9) and 1.2 (95% CI: 1.0–1.4) for therapies with unboosted and boosted PI, respectively. For the time to virological failure, multivariable HRs were 1.1 (95% CI: 0.6–2.0) and 0.6 (95% CI: 0.2–1.6) for unboosted and boosted PI therapies, respectively.

### Accumulation of Major PI Mutations

The accumulation of major PI mutations was not higher among patients detected with a minor PI mutation while ART-naïve. Of 63 patients who experienced a virological failure on first-line ART, 43 (68.3%) had a resistance test performed. 7/19 (36.8%) patients without minor PI mutations and 9/24 (37.5%) accumulated a major mutation, respectively (*P* = 1.000).

## Discussion

We found that the presence of minor PI mutations did not influence the virological outcome of first-line ART in HIV subtype B infected individuals. The prevalence of some minor PI mutations was found to be very high. Therefore, it is of great value to know that these mutations did not exhibit a negative impact on therapy outcome. In our study, neither the time to viral suppression, nor the time to virological failure differed between patients with and without minor PI mutations. Moreover, the risk for the emergence of a major PI mutation was not increased.

Today, first-line ART often includes non-nucleoside reverse transcriptase inhibitors, especially in resource-limited settings. However, PIs may increasingly be needed as good alternatives, especially in the presence of transmitted drug resistance mutations which seem to be seriously on the rise in resource-limited settings [29]. Our findings disproved concerns that the high prevalence of minor PI mutations limits the use of PIs but it is to mention that we only focused on subtype B infections.

To our knowledge, this is the largest study analyzing the impact of minor PI mutations on treatment outcome. We were able to include 1199 treated patients from the highly representative dataset of the SHCS. Despite the large number of patients included, the sample size was too small to perform an analysis for some specific minor PI mutations. Therefore, we had to restrict the analysis to the most prevalent minor PI mutations and used the propensity score method. The regression adjustment with propensity scores is a good option when the number of exposed patients is large and the number of events small. This method has the advantage that the Cox regression only had to be adjusted for one co-variable, the propensity score. If too many variables are included in a regression model relative to the number of events, estimates can be incorrect [30]. However, for some specific minor PI mutations, the confidence intervals of the HRs were quite large, especially for the models studying time to virological failure. This indicates that the accuracy of some estimates is limited. Unfortunately, we lacked statistical power to compare different combinations of minor PI mutations. Although we found that the time to viral suppression was shorter for patients with 3 minor PI mutations compared to patients without mutations, we think that the small difference we observed has no clinical relevance or even may have occurred by chance, considering that the lower bound of 95% CI of the HR was very close to 1, namely 1.1.

Our study supports findings from previous smaller studies that found no negative impact of minor PI mutations on therapy outcome [16], [17], [18]. However, it stands in contrast with the so far largest published studies by Perno et al including 248 and 93 individuals, respectively [14], [15]. They found a higher risk of virological failure among patients with mutations at position 10 and 36 and a higher accumulation of major PI mutations. In contrast to our study, Perno et al. included different HIV-1 subtypes and they did not adjust their models for ethnicity. Ethnicity, however, is potentially an important confounder as it was found to be associated with treatment outcome in other studies [31], [32]. Furthermore, Perno et al. mainly studied ART regimens containing unboosted PIs whereas our sample mainly contains regimens with boosted PIs. However, our sensitivity analysis that exclusively included ART with unboosted PIs containing 255 patients also lacked evidence for an impact of minor PI mutations on treatment outcome.

We convincingly demonstrated that minor PI mutations have no effect on virological outcome in PI-containing first-line ART, at least in patients infected by HIV-1 subtype B.

## References

[1] Kantor R, Katzenstein DA, Efron B, Carvalho AP, Wynhoven B (2005). Impact of HIV-1 subtype and antiretroviral therapy on protease and reverse transcriptase genotype: results of a global collaboration.. PLoS Med.

[2] Vergne L, Peeters M, Mpoudi-Ngole E, Bourgeois A, Liegeois F (2000). Genetic diversity of protease and reverse transcriptase sequences in non-subtype-B human immunodeficiency virus type 1 strains: evidence of many minor drug resistance mutations in treatment-naive patients.. J Clin Microbiol.

[3] Servais J, Plesseria JM, Lambert C, Fontaine E, Robert I (2001). Genotypic correlates of resistance to HIV-1 protease inhibitors on longitudinal data: the role of secondary mutations.. Antivir Ther.

[4] Velazquez-Campoy A, Todd MJ, Vega S, Freire E (2001). Catalytic efficiency and vitality of HIV-1 proteases from African viral subtypes.. Proc Natl Acad Sci U S A.

[5] Holguin A, Paxinos E, Hertogs K, Womac C, Soriano V (2004). Impact of frequent natural polymorphisms at the protease gene on the in vitro susceptibility to protease inhibitors in HIV-1 non-B subtypes.. J Clin Virol.

[6] Kinomoto M, Appiah-Opong R, Brandful JA, Yokoyama M, Nii-Trebi N (2005). HIV-1 proteases from drug-naive West African patients are differentially less susceptible to protease inhibitors.. Clin Infect Dis.

[7] Vergne L, Paraskevis D, Vandamme AM, Delaporte E, Peeters M (2003). High prevalence of CRF02_AG and many minor resistance-related mutations at the protease gene among HIV-infected treatment-naive immigrants in Madrid.. AIDS.

[8] Vergne L, Stuyver L, Van Houtte M, Butel C, Delaporte E (2006). Natural polymorphism in protease and reverse transcriptase genes and in vitro antiretroviral drug susceptibilities of non-B HIV-1 strains from treatment-naive patients.. J Clin Virol.

[9] Rhee SY, Kantor R, Katzenstein DA, Camacho R, Morris L (2006). HIV-1 pol mutation frequency by subtype and treatment experience: extension of the HIVseq program to seven non-B subtypes.. AIDS.

[10] Rhee SY, Gonzales MJ, Kantor R, Betts BJ, Ravela J (2003). Human immunodeficiency virus reverse transcriptase and protease sequence database.. Nucleic Acids Res.

[11] Nijhuis M, Schuurman R, de Jong D, Erickson J, Gustchina E (1999). Increased fitness of drug resistant HIV-1 protease as a result of acquisition of compensatory mutations during suboptimal therapy.. AIDS.

[12] van Maarseveen NM, de Jong D, Boucher CA, Nijhuis M (2006). An increase in viral replicative capacity drives the evolution of protease inhibitor-resistant human immunodeficiency virus type 1 in the absence of drugs.. J Acquir Immune Defic Syndr.

[13] Shafer RW, Schapiro JM (2008). HIV-1 drug resistance mutations: an updated framework for the second decade of HAART.. AIDS Rev.

[14] Perno CF, Cozzi-Lepri A, Balotta C, Forbici F, Violin M (2001). Secondary mutations in the protease region of human immunodeficiency virus and virologic failure in drug-naive patients treated with protease inhibitor-based therapy.. J Infect Dis.

[15] Perno CF, Cozzi-Lepri A, Forbici F, Bertoli A, Violin M (2004). Minor mutations in HIV protease at baseline and appearance of primary mutation 90 M in patients for whom their first protease-inhibitor antiretroviral regimens failed.. J Infect Dis.

[16] Brumme CJ, Harrigan PR (2005). No inherent association between minor mutations in HIV protease at baseline and selection of the L90M mutation at the time of the first virological failure.. J Infect Dis 191: 1778–1779; author reply 1779–1780.

[17] Champenois K, Deuffic-Burban S, Cotte L, Andre P, Choisy P (2008). Natural polymorphisms in HIV-1 protease: impact on effectiveness of a first-line lopinavir-containing antiretroviral therapy regimen.. J Med Virol.

[18] Frater AJ, Beardall A, Ariyoshi K, Churchill D, Galpin S (2001). Impact of baseline polymorphisms in RT and protease on outcome of highly active antiretroviral therapy in HIV-1-infected African patients.. AIDS.

[19] Lataillade M, Chiarella J, Yang R, Schnittman S, Wirtz V (2010). Prevalence and clinical significance of HIV drug resistance mutations by ultra-deep sequencing in antiretroviral-naive subjects in the CASTLE study.. PLoS One.

[20] Thompson MA, Aberg JA, Cahn P, Montaner JS, Rizzardini G (2010). Antiretroviral treatment of adult HIV infection: 2010 recommendations of the International AIDS Society-USA panel.. JAMA.

[21] Schoeni-Affolter F, Ledergerber B, Rickenbach M, Rudin C, Gunthard HF (2010). Cohort profile: the Swiss HIV Cohort study.. Int J Epidemiol.

[22] von Wyl V, Yerly S, Boni J, Burgisser P, Klimkait T (2007). Emergence of HIV-1 drug resistance in previously untreated patients initiating combination antiretroviral treatment: a comparison of different regimen types.. Arch Intern Med.

[23] Johnson VA, Brun-Vezinet F, Clotet B, Gunthard HF, Kuritzkes DR (2010). Update of the drug resistance mutations in HIV-1: December 2010.. Top HIV Med.

[24] Cepeda MS, Boston R, Farrar JT, Strom BL (2003). Comparison of logistic regression versus propensity score when the number of events is low and there are multiple confounders.. Am J Epidemiol.

[25] Brookhart MA, Schneeweiss S, Rothman KJ, Glynn RJ, Avorn J (2006). Variable selection for propensity score models.. Am J Epidemiol.

[26] Weitzen S, Lapane KL, Toledano AY, Hume AL, Mor V (2005). Weaknesses of goodness-of-fit tests for evaluating propensity score models: the case of the omitted confounder.. Pharmacoepidemiol Drug Saf.

[27] Young J, Klein MB, Ledergerber B (2011). Noncirrhotic portal hypertension and didanosine: a re-analysis.. Clin Infect Dis.

[28] Rosenbaum PR, Rubin DB (1983). The Central Role of the Propensity Score in Observational Studies for Causal Effects.. Biometrika.

[29] Hamers RL, Wallis CL, Kityo C, Siwale M, Mandaliya K (2011). HIV-1 drug resistance in antiretroviral-naive individuals in sub-Saharan Africa after rollout of antiretroviral therapy: a multicentre observational study.. Lancet Infect Dis.

[30] Harrell FE, Lee KL, Califf RM, Pryor DB, Rosati RA (1984). Regression modelling strategies for improved prognostic prediction.. Stat Med.

[31] Ribaudo H, Smith H, Robbins G (2011). Race Differences in the Efficacy of Initial ART on HIV Infection in Randomized Trials Undertaken by ACTG. CROI.. Boston, USA.

[32] Staehelin C, Keiser O, Calmy A, Weber R, Elzi L (2011). Longer term clinical and virological outcome of Sub-Saharan African participants on antiretroviral treatment in the Swiss HIV Cohort Study.. J Acquir Immune Defic Syndr.

